# Metabolomic dynamics of the arsenic-transformed bronchial epithelial cells and the derived cancer stem-like cells

**DOI:** 10.7150/ijbs.67314

**Published:** 2022-01-01

**Authors:** Yao Fu, Zhuoyue Bi, Lingzhi Li, Priya Wadgaonkar, Yiran Qiu, Bandar Almutairy, Wenxuan Zhang, Akimasa Seno, Chitra Thakur, Fei Chen

**Affiliations:** 1 Stony Brook Cancer Center, Renaissance School of Medicine, The State University of New York, Stony Brook University, Lauterbur Drive, Stony Brook, NY 11794, USA.; 2 Department of Pharmaceutical Sciences, Eugene Applebaum College of Pharmacy and Health Sciences, Wayne State University, 259 Mack Avenue, Detroit, MI 48201, USA.; 3 Department of Pathology, Renaissance School of Medicine, Stony Brook University, 101 Nicolls Road, Stony Brook, NY 11794, USA.

**Keywords:** arsenic, cancer, metabolomics, metabolism, cancer stem cells

## Abstract

Accumulating evidence indicates a carcinogenic role of environmental arsenic exposure, but mechanisms on how arsenic fosters malignant transformation of the normal cells are not fully established. By applying untargeted global metabolomics approach, we now show that arsenic is highly capable of perturbing the intracellular metabolic programs of the human bronchial epithelial cells, some of which are prominent hallmarks of cancer cell metabolism. To understand the spatiotemporal patterns of arsenic regulation on multiple metabolic pathways, we treated the cells with environmentally relevant concentration of arsenic, 0.25 μM, consecutively for 6 weeks to 24 weeks, and found that arsenic prompted heme metabolism, glycolysis, sphingolipid metabolism, phospholipid catabolism, protein degradation, and cholesterol breakdown constitutively, but inhibited metabolism of uracil-containing pyrimidine, carnitine, serotonin, polyamines, and fatty acid β-oxidation. A strong inhibition of all metabolites in mitochondrial tricarboxylic acid (TCA) cycle was noted in the cells treated with As^3+^ for 6 to 13 weeks. However, the metabolites in the earlier, but not the later steps of TCA cycle, including citrate, aconitate and isocitrate, were induced at 16 weeks through 24 weeks of arsenic treatment. This comprehensive metabolomics analysis provides new insights into metabolic perturbation by arsenic and may lead to more precise indications of arsenic in molecular carcinogenesis.

## Introduction

Arsenic is arguably the first suspected human carcinogen that was identified by the mystic and philosophical physician Paracelsus during the Renaissance 1. In his observational study of “mala metallorum” that was believed to be lung cancer of miners nowadays, Paracelsus hypothesized that sustained exposure to arsenic-containing dust or air from mining and processing of natural ores is the major cause of pulmonary symptoms that can rapidly progress to cachexia 2. As a naturally occurring and one of the most abundant elements of the Earth's crust, arsenic can be found in water, air, food, and soil. The most common source of environmental arsenic exposure is the drinking water contamination due to the leaching of arsenic from rocks and soil 3. In addition, emerging evidence suggests that some agricultural products, including rice, fruits and certain vegetables, are enriched with arsenic due to their cultivation in fields with high levels of arsenic in the soil or irrigation water 4. It is generally accepted that organic forms of arsenic are less harmful, whereas the inorganic form, especially the trivalent form of arsenic, As^3+^, causes some major health issues 5. Well-documented epidemiological and case-control studies in the United States, Bangladesh, Chile, China, Vietnam, Taiwan, India, and Mexico provided unequivocal evidence indicating association of increased cancer incidence rates with long-term arsenic exposure 3. These observations were further supported by the facts of carcinogenic effects of arsenic during the development of cancers in lung 6, liver 7, skin 8, bladder 9, breast 10, prostate 11, kidney 12, etc., in a number of experimental settings 13.

It remains to be fully understood how environmental exposure to arsenic causes malignant transformation of the normal cells. Unlike some chemical carcinogens that induce DNA damage and genetic mutation, several earlier studies revealed that arsenic itself is not a potent mutagen nor carcinogenic in rodent models 14. However, in human-hamster hybrid cells, studies by Hei et al. 15 unraveled that arsenic is highly capable of inducing chromosomal deletion, which possibly resulted from mitochondrial damage and ROS generation. Mitochondria function as the powerhouse of the cells by generating ATP and serving as a centralized hub in several metabolic networks, including glucose, fatty acid, amino acid, and the overflow of NAD+ and NADH. Accordingly, it is perceivable that any damages of the mitochondria induced by arsenic may lead to metabolic reprogramming of the cells 3. Indeed, our recent metabolomics analysis demonstrated a substantial inhibition of mitochondrial TCA cycle and a pronounced enrichment of the major metabolites in the glycolytic pathway in the cells treated with 0.25 μM As^3+^ for 1 to 3 days or consecutive treatment of the cells with 0.25 μM As^3+^ for 6 months 16. An overall enhancement of glycolysis was noted in most of the stem cells, cancer cells and cancer stem-like cells. It is believed that cancer cells use glycolysis, rather than TCA cycle, for fast ATP generation to meet the need of rapid growth of the cancer cells 17. More importantly, several glycolytic intermediators can be shunted into the pentose phosphate pathway for nucleotide biosynthesis, the hexosamine biosynthesis pathway for protein glycosylation, and serine-glycine pathway for protein synthesis and antioxidative capacity, all of which are essential for the stemness or proliferation of the cancer cells 3. The metabolic shift from mitochondrial TCA cycle to glycolysis, thus, may reflect a unique mechanism in mediating arsenic-induced carcinogenesis.

Despite these findings mentioned above, limited information is available on how arsenic regulates metabolism and how such a modulation in the metabolic dynamics affects cellular reprogramming with long-term arsenic exposure. To address this issue, in the present report, we treated human bronchial epithelial cells BEAS-2B with 0.25 μM As^3+^ consecutively for 6 to 24 weeks and evaluated the spatiotemporal metabolic patterns of amino acid, peptide, carbohydrate, energy, lipid, nucleotide, cofactors, and xenobiotics through a sensitive and quantitative untargeted global metabolomics using UPLC-MS/MS. Our data suggest that As^3+^ is able to modulate the biogenesis of few metabolites constitutively, whereas majority of metabolites showed transient induction or inhibition in a particular time period of As^3+^ treatment, which indicated specific characteristics of metabolism and metabolic adaptation of the cells at different stages of As^3+^-induced malignant transformation and/or the inception of the cancer stem-like cells.

## Results

### Time-dependent transformation of the As^3+^-treated BEAS-2B cells

**Figure 1:**
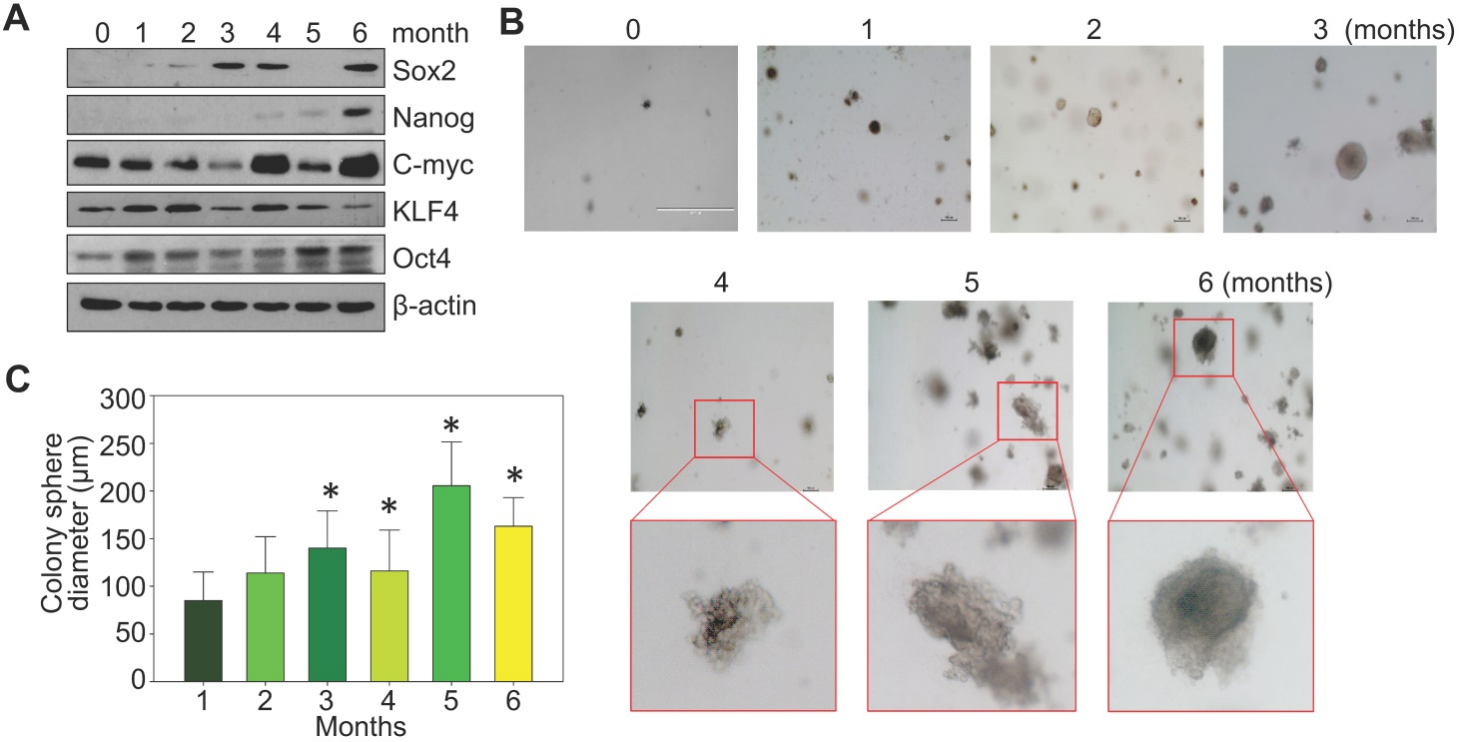
Consecutive As^3+^ treatment induces the transformation of the BEAS-2B cells. **A.** Western blotting with the indicated antibodies using protein extracts from the BEAS-2B cells treated with 0.25 μM As^3+^ for 1 to 6 months. **B.** Soft agar colony formation assay of the BEAS-2B cells treated with As^3+^ for the indicated times. **C.** Average diameters of the colony spheres of the cells treated with As^3+^ for the indicated times. Data show average diameters of randomly selected 15 colonies in each group.

BEAS-2B cells are immortalized but are noncancerous human bronchial epithelial cells. Under normal culture condition, these cells can be passaged but are unable to form notable cell colonies in soft-agar colony formation assay. To investigate a detailed time-course of arsenic-induced malignant transformation, these cells were consecutively treated with 0.25 μM arsenic (As^3+^) for 1 to 6 months, followed by Western blotting and soft-agar assay. In control cells, some basal expression of MYC (c-myc), KLF4 and OCT4 could be detected. A roughly time-dependent induction of MYC and OCT4 were observed in the cells treated with As^3+^ for 1 to 6 months (Fig. 1a). SOX2 induction occurred after 2-months of As^3+^ treatment. NANOG expression was not detected until the cells were treated with As^3+^ for 4 to 6 months. Except some clustered cell debris or tiny cell clusters, no cell colony formation was observed in the control cells and the cells treated with As^3+^ for 1 or 2 months (Fig. 1b). Notable cell colonies occurred in the cells treated with As^3+^ for 3 to 6 months, among which many colonies exhibited large sizes as measured by the colony diameters (Fig. 1c). These data, thus, clearly indicate that even for the immortalized BEAS-2B cells, As^3+^-induced transformation requires at least three months of consecutive treatment.

### Metabolomic characterization of the As^3+^-transformed cells

**Figure 2:**
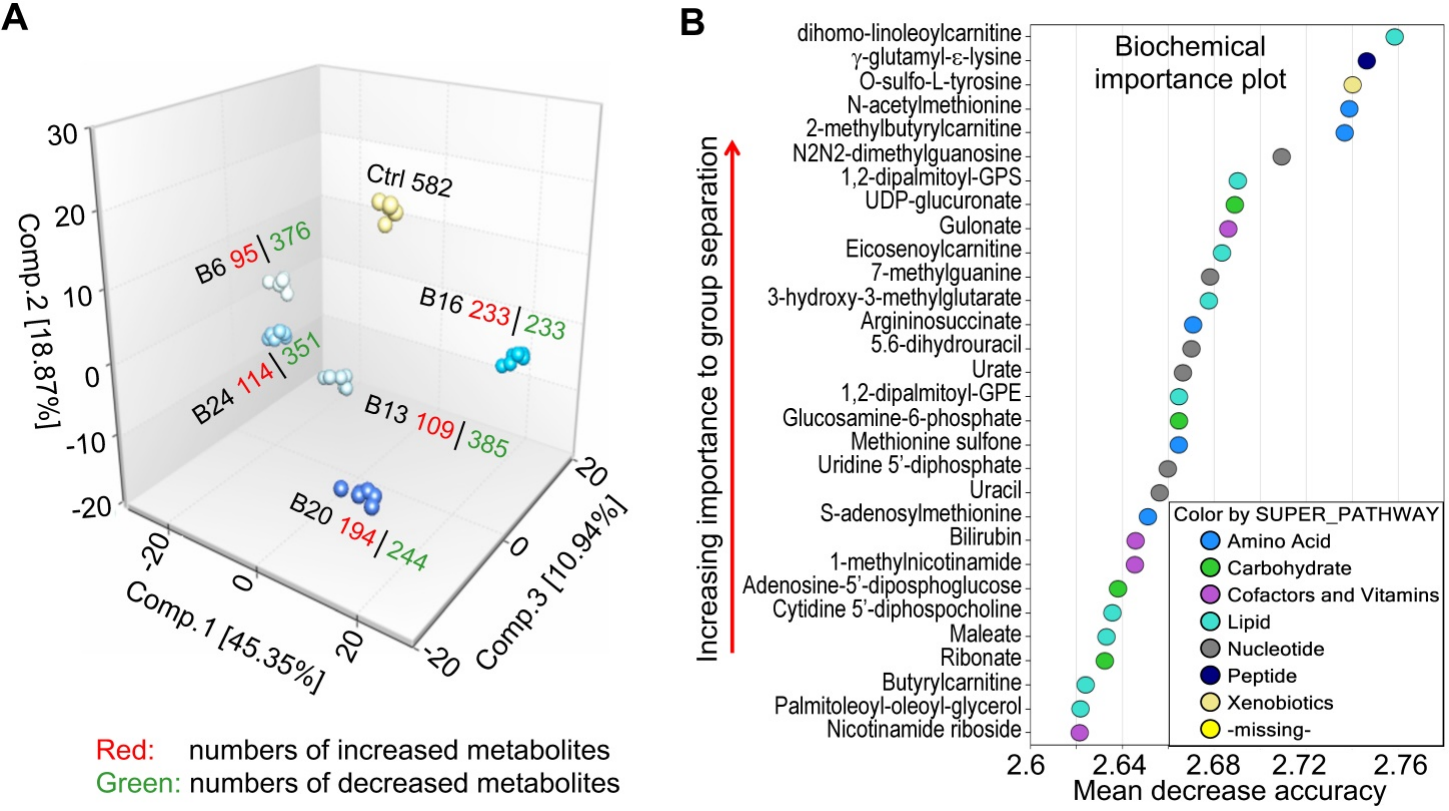
** Exploratory data analysis of untargeted global metabolomics. A.** Principal component analysis (PCA) through a mathematical procedure that can be used to obtain a high-level view of the structure of the metabolomics dataset. Ctrl: control; B6-24: BEAS-2B cells treated with 0.25 μM As^3+^ consecutively for 6 to 24 weeks. **B.** Random forest (RF) classifier of the metabolomics dataset using a meta estimator that fits a number of decision tree classifiers on the entire dataset as well as different As^3+^ treatment groups.

We had previously characterized As^3+^-induced cancer stem-like cells (CSCs) through metabolomics profiling 16. However, the CSCs in that study were derived from selected colonies of the transformed cells induced by consecutive treatment of the BEAS-2B cells with 0.25 μM As^3+^ for 6 months, which may be non-representative for the entire population of the transformed cells neither biochemically nor epigenetically 18, 19. To address this point, we treated BEAS-2B cells with 0.25 μM As^3+^ for 6 to 24 weeks and collected the entire cell population at each time point for untargeted metabolomics study. The metabolomics dataset comprises a total of 584 compounds of known identity (named biochemicals). Following normalization to Bradford protein concentration, log transformation and imputation of missing values, if any, with the minimum observed value for each compound, ANOVA contrasts were used to identify biochemicals that differed significantly between experimental groups. A summary of the numbers of biochemicals that achieved statistical significance (p≤0.05, 582 metabolites), as well as those approaching significance (0.05<p<0.10, 2 metabolites), is indicated in Fig. 2a.

### Principal component, random forest and statistical overview

A mathematical procedure was used for Principal Component Analysis (PCA) to obtain a high-level view of the structure of a dataset. Briefly, PCA permits visualization of how individual samples in a dataset differ from each other. Samples with similar biochemical profiles cluster together, whereas samples with different biochemical profiles segregate from one another. As such, this analysis aids in determining if the different experimental groups can be segregated based on differences in their overall metabolic signature. As shown in Fig. 2a, the samples were clustered closely together within groups (n = 6) and were well-separated among groups, suggesting little sample variation and that the groups exhibited different biochemical signatures. Notably, every experimental group displayed a distinct global biochemical profile. Treatment of the cells with As^3+^ for a relatively short time period (6- and 13-weeks) led to migration of samples in component 1 and 2 relative to baseline, while exposure for longer periods of time (16- and 20-weeks) led to the migration back towards baseline in component 1. Notably, the longest As^3+^ exposure (24 weeks) was more closely related to the 6- and 13-weeks exposures than the 16- and 20-weeks exposures, which could potentially reflect a biochemical adaptation to long-term As^3+^ exposure.

Random forest (RF) is an unbiased and supervised classification technique based on an ensemble of a large number of decision trees that attempts to bin individual samples in groups based on their metabolite similarities and differences. When RF analysis was performed comparing baseline cells (control) to the combined five As^3+^-treated groups, the results provided a predictive accuracy of 100% which was well-above random chance (50% for two groups), suggesting the list of top 30 biochemicals involved in separating the groups could underlie their phenotypic differences (Fig. 2b). The metabolites showing the strongest predictive power were primarily involved in the metabolism of lipids, nucleotides, and amino acids, indicating these pathways may be important for distinguishing baseline from As^3+^-treated cells.

Comparison of global biochemical profiles for As^3+^-treated BEAS-2B cells revealed several key metabolic differences as highlighted below. There were numerous statistically significant changes observed in each comparison. For example, 85% (494/584) of the biochemicals detected at the B13 time point displayed significant changes relative to baseline. Accordingly, the discussion will focus primarily on the most profound biochemical changes relative to baseline. With the exception of the B16 time point, the majority of biochemicals underwent significant decreases relative to baseline, which may reflect a general decrease or slowing of metabolic activity and decreased cell growth or proliferation during As^3+^ exposure.

### Few metabolites showed persistent induction or inhibition by As^3+^

**Figure 3:**
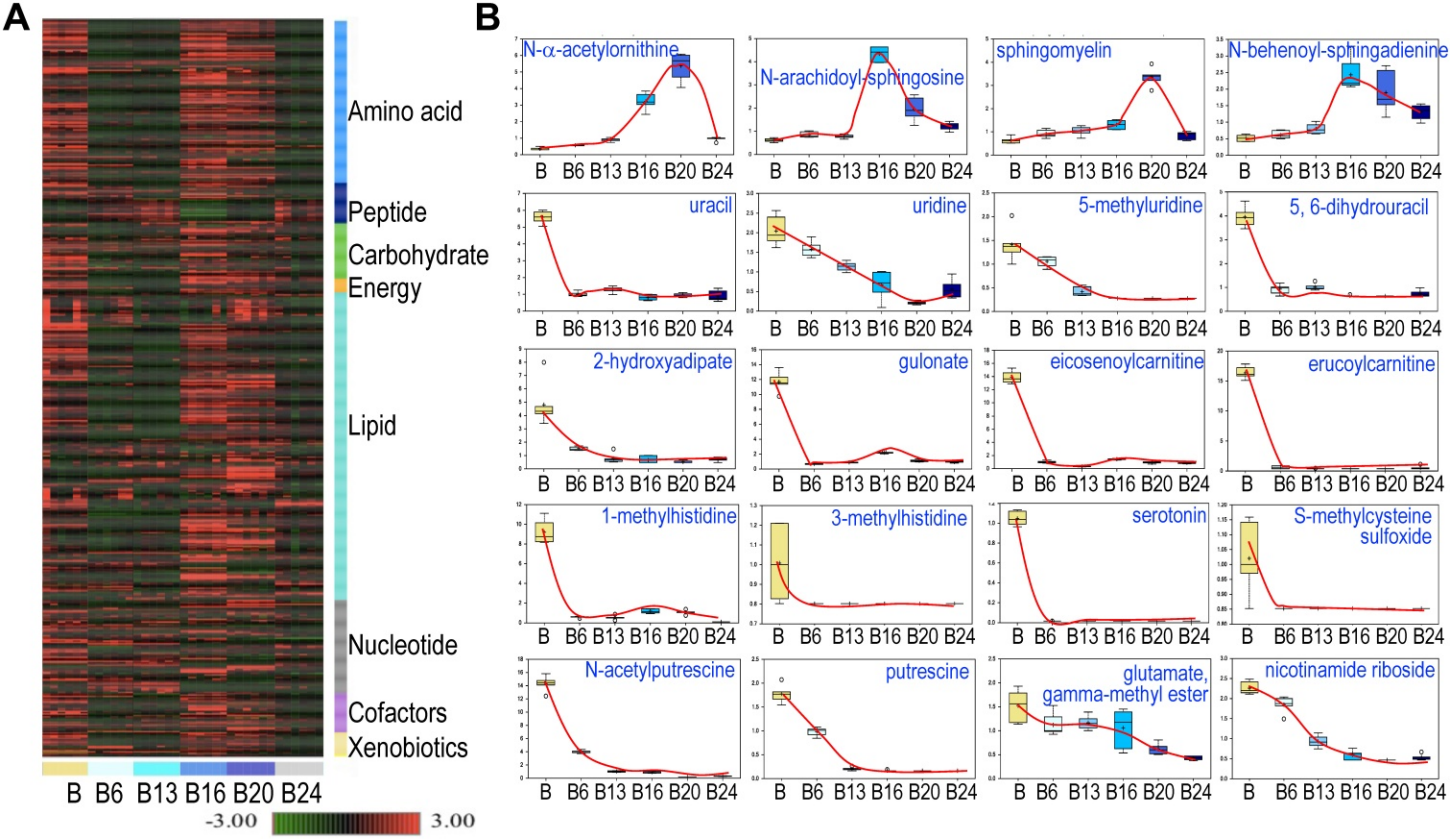
** Spatiotemporal dynamics of metabolism among the control cells and the cells treated with 0.25 μM As^3+^ for 6 to 24 weeks (B6-24). A.** Heatmap of the expression levels of 584 metabolites in control cells and the cells treated with 0.25 μM As^3+^ for 6 to 24 weeks. Six replicates were used for each group of the cells. **B.** Expression dynamics of the indicated individual metabolites. Top roll shows metabolites that were sustainedly induced with different degree at each time point of As^3+^ treatment. Other rolls show metabolites that were substantially inhibited by As^3+^ at every time point. Y- and X-axis represent the relative level of metabolomics quantification and time points of 0.25 μM As^3+^ treatment of the cells, respectively.

**Figure 4:**
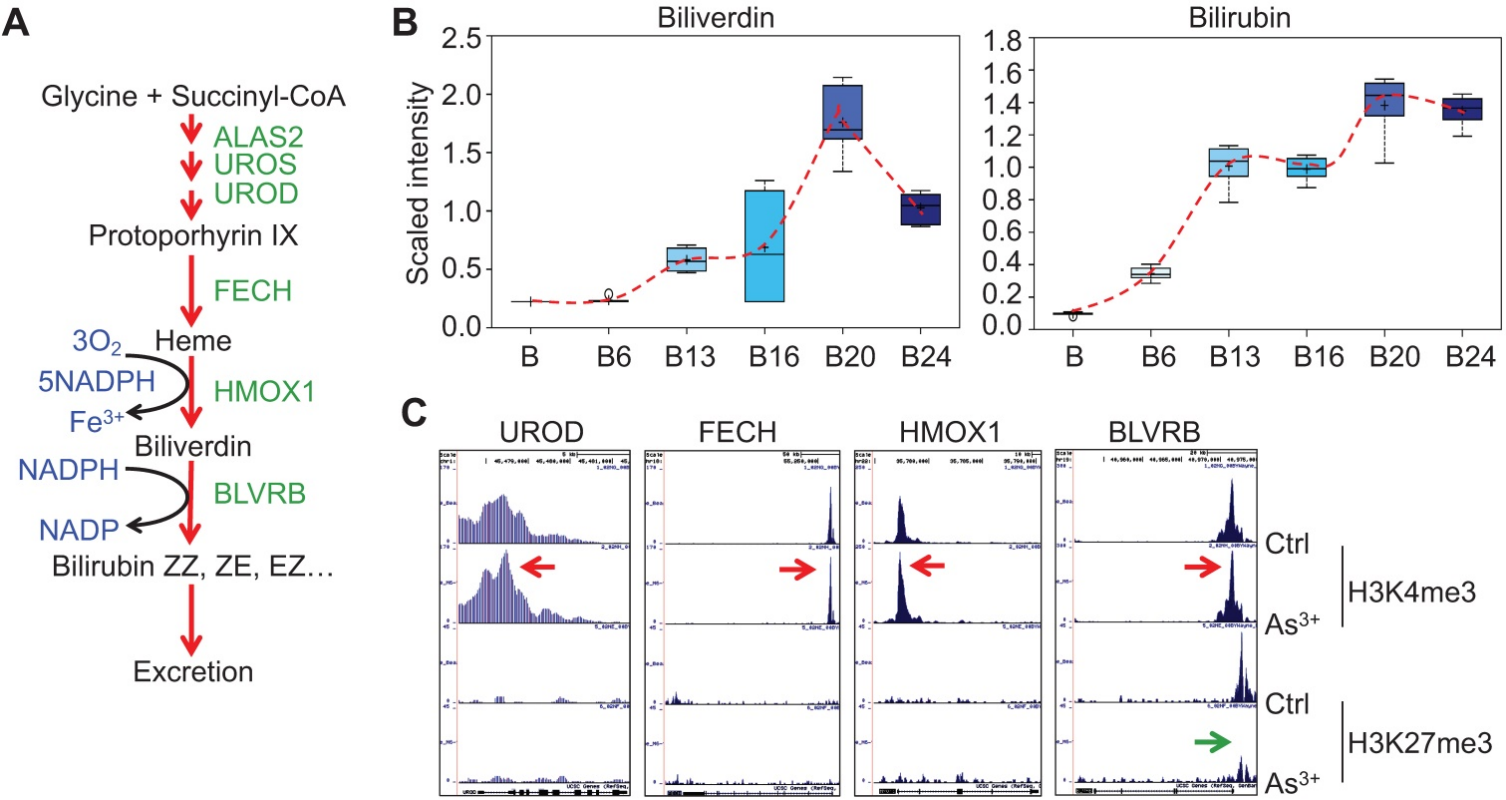
** As^3+^ promotes heme metabolism. A.** Schematic diagram of heme metabolic pathway. Enzymes involved in each step of the heme metabolism are indicated with green color. **B.** Actual levels of biliverdin and bilirubin in each group of the cells, which showed a time-dependent induction by 0.25 μM As^3+^. **C.** ChIP-seq profile of the genes encoding the indicated rate-limiting enzymes for heme metabolism in control cells and the cells treated with 0.25 μM As^3+^ for 24 weeks. Red arrows indicated an enhanced enrichment of the active epigenetic marker H3K4me3 on these genes in response to As^3+^, and green arrow denoted reduced repressive epigenetic marker H3K27me3 in the indicated gene in response to As^3+^.

**Figure 5:**
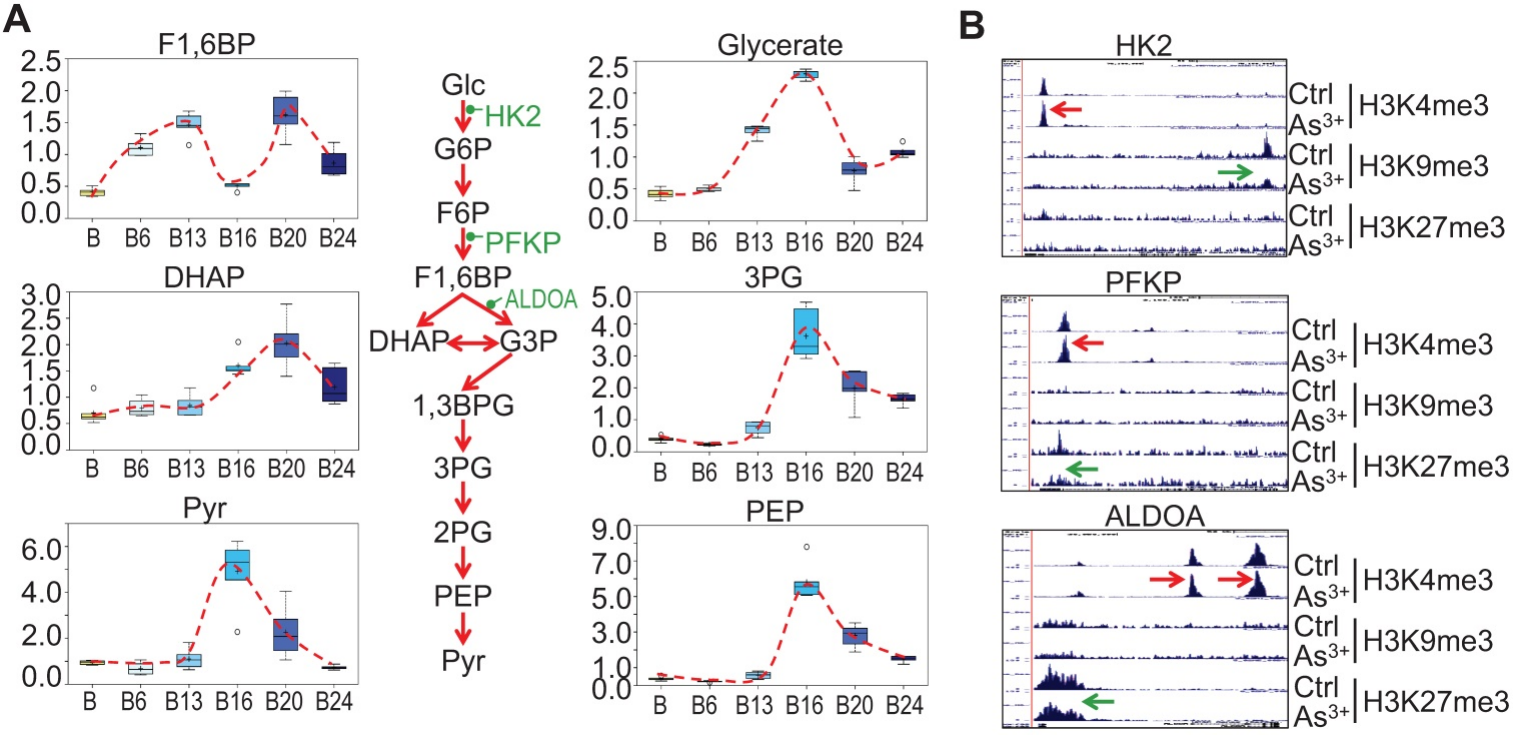
** Glycolytic features of the As^3+^-treated cells. A.** Relative quantification of the indicated glycolytic intermediates as determined by metabolomics in control cells and As^3+^-treated cells. **B.** Genome browser screenshots of ChIP-seq for the selected glycolytic genes. Red arrows indicated enhanced active epigenetic marker H3K4me3; green arrows indicated reduced level of repressive epigenetic marker H3K9me3 or H3K27me3.

Hierarchical Clustering Analysis of the detected metabolites was generally consistent with the PCA, showing perfect clustering of samples within groups and no overlap between groups (Fig. 3a). In general, there is an initial suppression of metabolism by As^3+^ treatment for 6 to 13 weeks, followed by a pronounced recovery among the cells treated with As^3+^ for 16 to 20 weeks. At 24 weeks of As^3+^ treatment, a clear decline of many metabolites was noted, which indicates selections and metabolic adaptation of the cell population under the influence of consecutive As^3+^ treatment. Among the detected 584 metabolites, 456 (78%) showed either induction or inhibition at certain time point(s) of As^3+^ treatment. There are 109 (19%) metabolites showed persistent inhibition at every time point and 19 (3%) metabolites exhibited steady induction at every time point of As^3+^ treatment. The sustainedly induced metabolites by As^3+^ include N-α-acetylornithine, some sphingolipids (Fig. 3), heme metabolites (Fig. 4b), glycolytic products fructose 1,6-bisphosphate and glycerate (Fig. 5), plasmalogen 1-(1-enyl-stearoyl)-2-oleoyl-GPE, and membrane metabolite 1-stearoyl-2-oleoyl-GPG (data not shown). It is interesting to note that As^3+^ is highly potent in inhibiting the metabolism of uracil-containing pyrimidine, carnitine, methylhistidine, serotonin, polyamines, nicotinamide riboside, and other (Fig. 3b).

### As^3+^ enhances heme metabolism

Several enzymes, including ALAS2, UROS, UROD, etc, are involved in the heme synthesis in mitochondria through condensation of succinyl-CoA with the amino acid glycine (Fig. 4a). As^3+^ exposure is known to induce the mitochondrial enzyme heme oxygenase 1 (HMOX1) that catalyzes the degradation of heme into biliverdin and, ultimately, bilirubin. Consistent with this notion, As^3+^ treatment led to marked elevation in biliverdin and bilirubin at nearly every time point relative to baseline (Fig. 4b). These changes may reflect increased mitochondrial stress, which is known to induce HMOX1 expression in response to metal exposure. Meanwhile, ChIP-seq showed a notably enhanced enrichment of H3K4me3, an active transcription marker, on the genes of UROD, FECH, HMOX1, and BLVRB that catalyze heme metabolism in the cells treated with As^3+^ for 24 weeks, along with a reduction of H3K27me3, a repressive epigenetic marker, on the BLVRB gene (Fig. 4c).

### Strengthened glycolysis by As^3+^

Our previous studies on the As^3+^-induced cancer stem-like cells (CSCs) suggested that As^3+^ induces metabolic shift of glucose from mitochondrial TCA cycle to glycolysis 16. Partially consistent with this observation, longer-term As^3+^ exposure, 16 to 24-weeks, led to significantly elevated 3-carbon glycolytic intermediates including glycerate, 3-phosphoglycerate (3PG), phosphoenolpyruvate (PEP), and pyruvate (Pyr) (Fig. 5a) with a peak at 16 weeks. Increases in 3-carbon glycolytic intermediates are associated with declined channeling of glycolysis to TCA cycle in mitochondrial, and may contribute to an energy starved metabolic state. It is very likely that this overall enhancement of glycolysis may be resulted from the gain of H3K4me3 and diminishment of the repressive markers H3K9me3 or H3K27me3 on the rate limiting genes, such as HK2, PFKP and ALDOA, in As^3+^-treated cells (Fig. 5b).

### A unique pattern of mitochondrial TCA cycle in response to As^3+^ exposure

**Figure 6:**
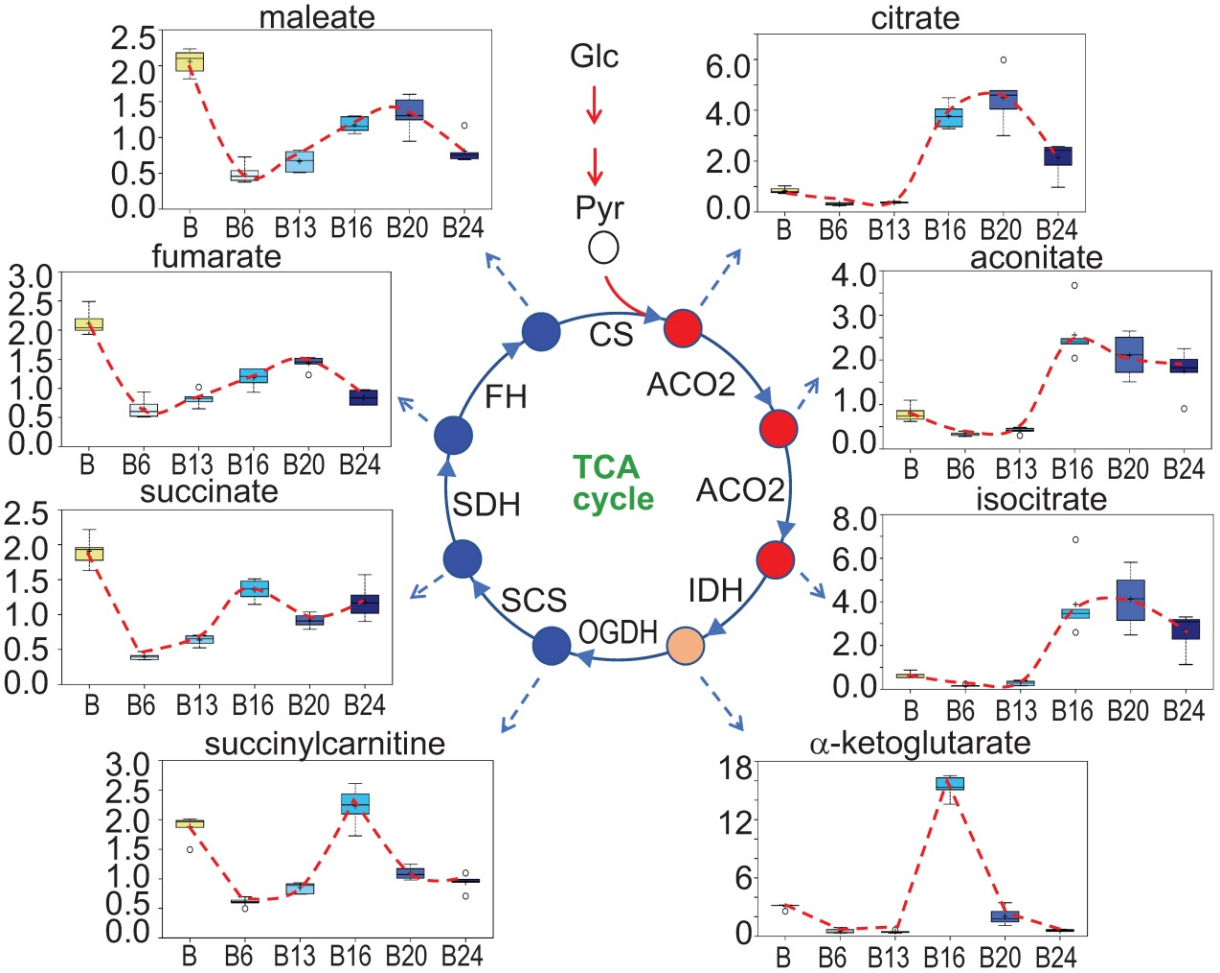
** Perturbance of TCA cycle by As^3+^.** Panels show spatiotemporal pattern of intermediates in TCA cycle in the cells treated with As^3+^ for different time periods.

In As^3+^-induced CSCs that were derived from a single colony of the transformed cells featured with asymmetrical division and overexpression of the stemness genes, a substantial inhibition on mitochondrial oxidative phosphorylation and TCA cycle was noted 3, 16. In the entire population of cells treated with 0.25 μM As^3+^ for 6 to 24 weeks, however, a unique pattern of the TCA cycle was observed. There is a strong inhibition of all metabolites in TCA cycle when the cells were treated with As^3+^ for 6 and 13 weeks. Interestingly, at 16 weeks of As^3+^ treatment, citrate, aconitate and isocitrate showed a significant increase until 24 weeks, which may reflect an adaptive or compensatory process in the earlier steps of TCA cycle. α-ketoglutarate and succinylcarnitine increased at 16-weeks treatment but declined to the levels of 6- and 13-weeks at 20 and 24 weeks. For the metabolites of succinate, fumarate and maleate, despite some marginal recovery at 16-weeks of As^3+^ treatment after the initial inhibition at the time points of 6- to 13-weeks, the levels of these metabolites are still much lower than the control cells, suggesting As^3+^ caused a sustained inhibition on the later steps of TCA cycle (Fig. 6).

### Enhanced potential of DNA synthesis in cellular response to As^3+^

**Figure 7:**
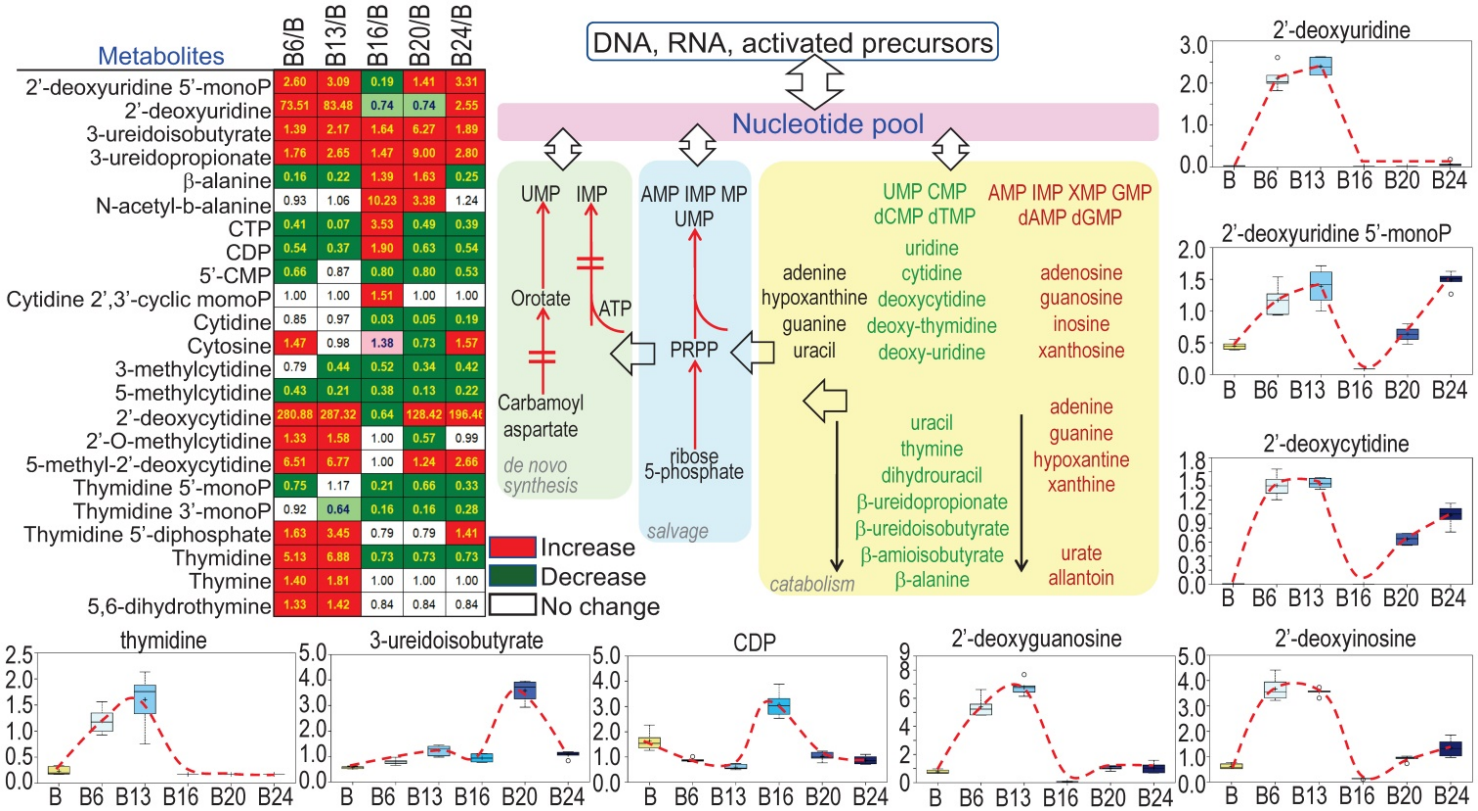
** As^3+^ strengthens biosynthesis of nucleotides.** The heatmap/table shows quantitative ratios between As^3+^-treated cells and the control cells of the indicated nucleotide precursors and nucleotides. Schematic diagram summarized sub-pathways of nucleotide biosynthesis. Additional panels show the actual levels of several selected metabolites in nucleotide metabolic pathway in control cells and the cells treated with 0.25 μM As^3+^ for 6 to 24 weeks.

Sustained growth and proliferation of the malignantly transformed cells and cancer cells are highly dependent on the *de novo* synthesis of nucleotides to support fast replication of DNA. It is not surprising, therefore, As^3+^ treatment increased the levels of most of the precursors of nucleotides (Fig. 7), esp. the multiple deoxynucleosides including 2'-deoxycytidine, 2'-deoxyuridine, 2'-deoxyguanosine, and 2'-deoxyinosine. In contrast to the metabolites in TCA cycle that showed inhibition by As^3+^ at 6 and 13 weeks, most of these deoxynucleosides were dramatically elevated at 6- and 13-weeks of As^3+^ treatment, which correlated to the metabolic shift from TCA cycle to glycolysis that shunted to the pentose phosphate pathway for the biosynthesis of nucleotides. The elevated deoxynucleosides were accompanied by consistently elevated 3-uredioisobutyrate, 3-ureidopropionate and thymidine. Together, these changes may reflect increased synthesis and degradation of DNA, RNA and nucleotide catabolism potentially related to cell transformation and DNA damage.

### As^3+^ promotes protein turnover and degradation

**Figure 8:**
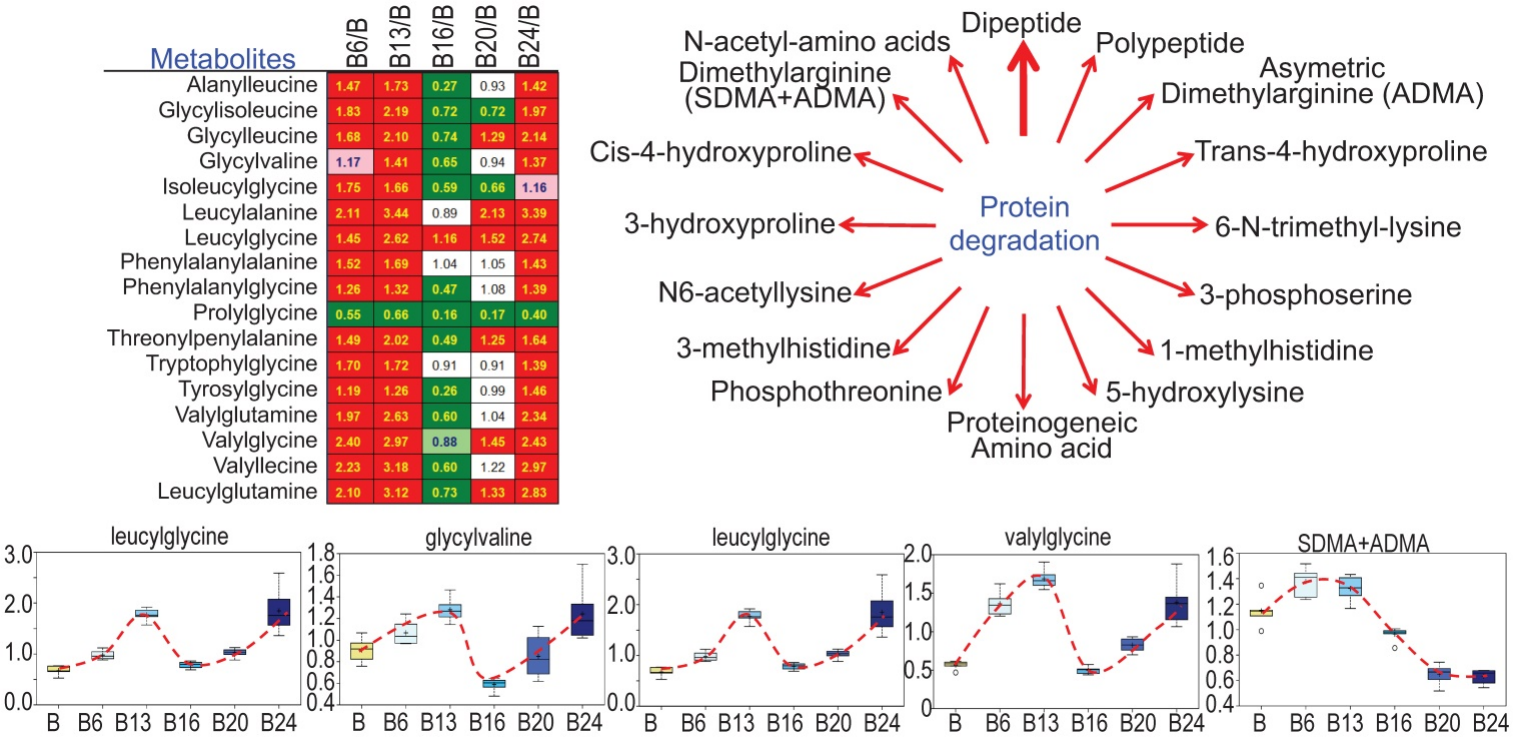
** Increased levels of dipeptides in the As^3+^-treated cells.** Except prolylglycine, all other listed dipeptides were increased by As^3+^ treatment for 6 to 13 weeks. Diagram shows major metabolites produced during protein degradation. Bottom panels show the quantitative levels of these indicated dipeptides in control cells and the cells treated with As^3+^ for the different time periods.

Cachexia is a common clinical sign for cancer patients due to hypercatabolism of proteins, which was first described as “wasting disease of miners” by Paracelsus 1. It is estimated that 50 - 80% of cancer patients are affected by cachexia featured with body weight loss, muscle wasting, adipose tissue depletion, and metabolic abnormalities 20. Such protein metabolic pattern is largely driven by overactivation of multiple proteolytic systems, such as ubiquitin-proteasome, lysosome leakage, autophagy and endoplasmic reticulum (ER) stress. In response to As^3+^ treatment, nearly every dipeptide species was elevated (Fig. 8), which indicates an increase in protein degradation and/or turnover induced by As^3+^. Interestingly, all of these dipeptides showed a bi-phase induction by As^3+^ with a feature of significant induction at 6 and 13 weeks while returning to basal level at 16 weeks, and further showing an induction again at 20 and 24 weeks.

### As^3+^ elicits a distinctive paradigm of lipid metabolism

**Figure 9:**
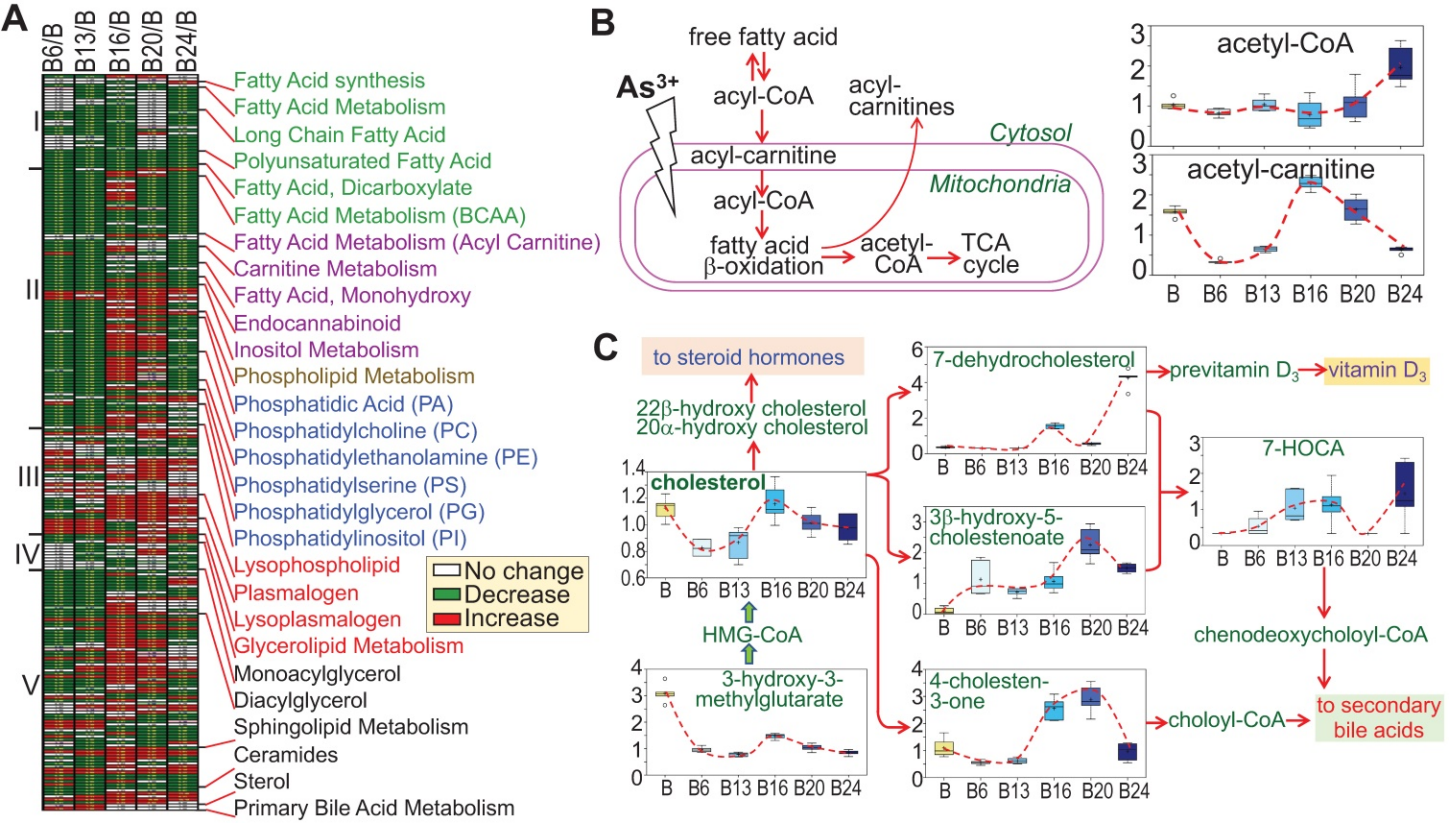
** Divergent metabolic patterns of lipids in cellular response to As^3+^. A.** Heatmap shows ratios of 255 lipid metabolites in As^3+^-treated cells vs control cells. The Roman numbers on the left of heatmap indicated arbitrary classifications of lipids based on their expression levels in the cells treated with As^3+^ for 6 to 24 weeks. The identities of lipid sub-family were listed on the right of the heatmap. **B.** Diagram of fatty acid β-oxidation in mitochondria along with the quantitative levels of acetyl-CoA and acetyl-carnitine. **C.** Diagram of cholesterol biosynthesis and catabolic pathway along with quantification of the indicated metabolites in this pathway.

There is evidence indicating metabolic disorders, such as insulin resistance and type2 diabetes, in individuals who were exposed to environmental As^3+^ 21, 22. Furthermore, Kuo et al. 23 in a 15-year birth cohort study in Taiwan revealed that early life As^3+^ exposure promoted atherogenic lipid metabolism characterized by higher LDL and non-HDL levels in adolescence. In the cells treated with As^3+^, we indeed noted some unique features of lipid metabolism. Based on the pattern of induction or inhibition in response to As^3+^ treatment for different time periods, we arbitrarily divided 245 lipids into five subgroups based on the expression pattern at different time points (Fig. 9a). Group I comprised with metabolites in fatty acid synthesis/metabolism, biosynthesis of long chain, polyunsaturated, and dicarboxylate fatty acid, which are generally inhibited by As^3+^ at every time point, possibly indicating deficiency of β-oxidation of fatty acid due to mitochondrial dysfunction induced by As^3+^ (Fig. 9b). Group II contains acyl carnitine derivatives and membrane remodeling metabolites, which exhibited a significant decrease in As^3+^-treated cells at shorter time points, 6 and 13 weeks, but transiently increased with a continued As^3+^ exposure for 16 weeks, followed by a predominant decrease at 24 weeks of As^3+^ exposure. These findings are generally consistent with a prior study of short-term As^3+^ exposure in a gastric carcinoma cell line 24. In addition, As^3+^-treated cells exhibited significantly reduced levels of the phospholipid precursors choline, choline phosphate, and diacylglycerols as well as most phosphatidylcholines, phosphatidylethanolamines, phosphatidylserines, and phosphatidylinositols at shorter time points (there were some increases at later time points). These changes were accompanied by increases in the phospholipid breakdown products glycerophosphoethanolamine and some lysophospholipids, as indicated in group III, some of which showed increase at every time points of As^3+^ treatment. Treatment of the cells with As^3+^ showed no or limited inhibitory effect on the lipids in group IV that contains metabolites of monoacylglycerol. In group V, some of the sphingolipid metabolites and ceramides showed early induction by As^3+^ until to the later time point. Another sub-group in group V is the sterol family for cholesterol breakdown, including 3-β-hydroxy-5-cholestanoate, and 7-α-hydroxy-3-oxo-4-cholestanoate (7-HOCA), were markedly elevated with As^3+^ exposure. In addition, cholesterol and its precursor 3-hydroxy-3-methylglutarate were significantly reduced at most time points (Fig. 9c). Collectively, these data clearly demonstrated that As^3+^ is able to reduce fatty acid oxidation as well as membrane remodeling, but promote cholesterol catabolism.

### Potential association of As^3+^-provoked sphingolipid metabolism with human cancers

**Figure 10:**
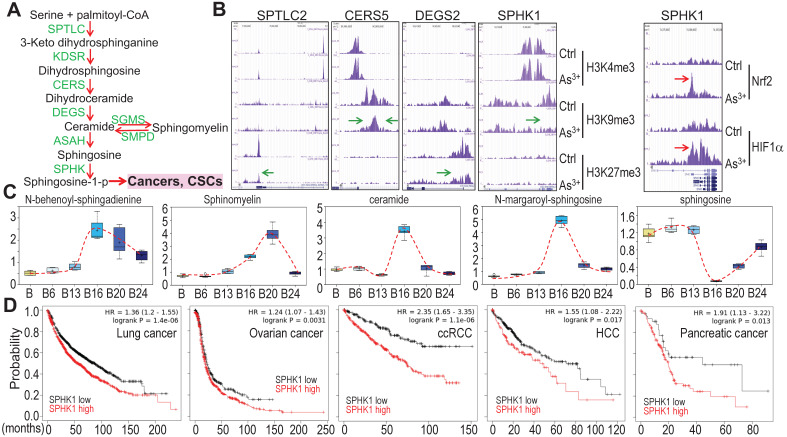
As^3+^ potentiates sphingolipid metabolism. **A.** Simplified sphingolipid metabolic pathway. **B.** ChIP-seq showed reduced enrichment of repressive epigenetic marker H3K9me3 or H3K27me3, as denoted by green arrows, on the indicated sphingolipid metabolic genes in the cells treated with 0.25 μM As^3+^ for 24 weeks. Right panel showed enhanced enrichment of Nrf2 and HIF1α on the SPHK1 gene in the cells treated with 1 μM As^3+^ for 6h. Green arrows indicate reduced level of H3K9me3 or H3K27me3; Red arrows indicate increased level of Nrf2 or HIF1α on SPHK1 gene induced by As^3+^. **C.** Quantification of the selected sphingolipids by metabolomics among the control cells and cells treated with As^3+^ for 6 to 24 weeks. **D.** Higher expression of SPHK1 is associated with poorer survival of human lung cancer, ovarian cancer, clear cell renal cell carcinoma (ccRCC), hepatocellular carcinoma (HCC), and pancreatic cancer.

Growing evidence suggests strong association of altered sphingolipid metabolism with human cancer 25. A direct contribution of the metabolic product, sphingosine-1-phosphate (sphingosine-1-p), from this pathway to cancer stem cells (CSCs) (Fig. 10a) had also been reported 25, 26, which is consistent with the overexpression of sphingosine kinase 1 (SPHK1) in some types of cancer 27. ChIP-seq analysis revealed that consecutive As^3+^ treatment for 24 weeks reduced enrichment of the repressive histone H3 methylation marker H3K9me3 or H3K27me3 on the genes involved in several steps of sphingolipid metabolism (Figs. 10a and 10b), suggesting upregulation of these genes by As^3+^. We also observed a strong enrichment of Nrf2 and HIF1α on the SPHK1 gene in the BEAS-2B cells treated with 1 μM As^3+^ for 6h (Fig. 10b, right panel). This notion is supported by the increased levels of the key metabolites in this pathway in the cells treated with As^3+^ for 20 to 24 weeks (Fig. 10c). Since SPHK1 is the key rate limiting enzyme converting ceramide/sphingosine to sphingosine-1-p and increased expression of SPHK1 in some human cancers, we next surveyed the prognostic value of SPHK1 for some common human cancers using the public datasets of cancer patient survival (Kaplan-Meier plotter). There is a clear correlation between higher level of SPHK1 and poorer overall survival of the patients with lung cancer, ovarian cancer, clear cell renal cell carcinoma (ccRCC), hepatocellular carcinoma (HCC), and pancreatic cancer (Fig. 10d). All of these data, thus, point to the fact that rewired sphingolipid metabolism is one of the carcinogenic mechanisms associated with environmental As^3+^ exposure.

## Discussion

Environmental exposure to arsenic is still a major issue of public health globally. Well-established evidence unequivocally suggests that arsenic, especially, the inorganic trivalent form of arsenic, As^3+^, is a human carcinogen 3. The human exposure to environmental arsenic is a long-term process, usually several years to decades. The maximum allowable level of inorganic arsenic in drinking water in the US and some other parts of the world is 10 ppb. However, the arsenic level is reached to hundreds to thousands ppb in some water bodies, for example, the arsenic levels in continental surficial water in San Luis Potosi of Mexico is up to 8,684 ppb and in groundwater in Michoacan of Mexico is up to 1.506 million ppb 28. Despite extensively studied on the mechanism of carcinogenesis induced by As^3+^, limited information is available on whether and how As^3+^ exposure affects the intracellular metabolic programs that are linked to malignant transformation and the generation of CSCs. In our previous studies, we used 0.25 μM (~18 ppb) As^3+^ to treat the BEAS-2B cells consecutively for 3 to 6 months to mimic human exposure to environmental arsenic, and found the generation of CSCs featured with higher level of glycolysis and compromised mitochondrial TCA cycle 16, 18, 19. To extend these observations, in the current report we established a detailed long-term time course of 0.25 μM As^3+^ treatment and investigated the spatiotemporal patterns of metabolic pathways in these cells through untargeted metabolomics. Although the basal and induced expression of stemness transcription factors MYC, OCT4 and KLF4 can be detected in all of these time points of As^3+^ treatment, the expression of NANOG occurred only among the cells treated with As^3+^ for 4 to 6 months, which indicates that the transformation and generation of CSCs requires a longer time treatment of the cells. However, the overall metabolic changes can be detected in the cells treated with As^3+^ for days 16 to 24 weeks, suggesting that metabolic reprogramming occurs before the transformation and formation of the CSCs, or in other words, either malignant transformation or the establishment of CSCs requires metabolic reprogramming. Accordingly, there is significant interest in understanding how intracellular metabolism is disturbed by As^3+^.

Examination of the metabolomics data via PCA indicated strong clustering within sample groups and clear segregation among groups of the cells treated with As^3+^ for 6, 13, 16, 20, and 24 weeks, generally suggesting the different groups display different global metabolomic signatures. Indeed, all the relevant comparisons revealed at least 75% of the biochemicals detected were changing significantly relative to baseline (Fig. 2). The majority of biochemicals underwent significant decreases, indicating a broad slowing or impairment of metabolic processes in As^3+^-treated cells. In general, As^3+^ treatment caused metabolic changes consistent with an upregulation of heme metabolism, glycolysis, phospholipid catabolism, protein degradation, and cholesterol breakdown, but impaired metabolism of uracil-containing pyrimidine, carnitine, serotonin, polyamines, fatty acid β-oxidation, and mitochondrial TCA cycle. Interestingly, there was somewhat of a shift/recovery of early metabolomic changes at intermediate time points, possibly suggesting cellular metabolic adaptation or compensation to the effects of As^3+^. This potential adaptive process may have been accelerated through long-term passaging of cells and the influences of As^3+^ on epigenetics that determines the dynamics of gene expression, both of which could have been selected for cells partially resistant to As^3+^ exposure. Most of the metabolic shifts at intermediate time points faded by 24 weeks of As^3+^ exposure, which may reflect metabolic decompensation.

The findings in this report that As^3+^ promotes sphingomyelin biosynthesis (Figs. 3b and 10), phospholipid breakdown and cholesterol catabolism (Fig. 9) are in great agreement with the most recent human cohort study of Strong Heart Family and untargeted blood metabolomics by Sanchez et al 29 who showed that As^3+^ primarily affects fatty acid and lipid metabolism, such as glycerophospholipid and glycosphingolipid, and the methylated form of As^3+^ can also perturb energy metabolism in human subjects who were exposed to environmental As^3+^. Several earlier studies also suggested increased expression of the key enzymes in sphingolipid metabolism, such as SPHK1, in human cancers 25, and the direct contribution of the sphingolipid metabolic product, sphingosine-1-p, to the expansion of cancer stem-like cells 26. Thus, the capacity of As^3+^ in rewiring lipid metabolism may play key roles in the carcinogenesis associated with environmental As^3+^ exposure. Our previous study and current report also revealed an enhanced nucleotide metabolism due to the upregulation of glycolysis and the pentose phosphate pathway by As^3+^. This notion is partially consistent with the findings in a Chihuahua cohort study in Mexico that showed a positive association between drinking water arsenic exposure and increased purine levels of in urine 30.

The changes in the levels of metabolites in response to As^3+^ are indicative of the specific effects of As^3+^ on the related metabolic pathways. However, the interpretation of such data is confounded by the dynamics of the individual metabolic pathways that require the flow or flux of precursors and catabolic products. Thus, a stable isotope of metabolic flux is needed to trace the fate of some metabolites, especially the intermediates in glycolysis and TCA cycle through measuring the mass isotopologue patterns of the interested metabolites. In our previous single time point study, we did observe a significant increase in flux from glucose into the glycolytic intermediates in the As^3+^-induced CSCs 16.

The data presented to our knowledge provide the most comprehensive metabolic profile through metabolomics analysis of As^3+^ action in cell transformation and generation of the CSCs, some of which are well-aligned with the findings of the most recent human cohort studies of drinking water As^3+^ exposure 29. With such a deeper metabolomic and biochemical understanding, we anticipate that these results will inform the design of new preventive strategies of cancers associated with environmental exposure as well as provide new insights into specific designs of therapies for the cancers either associated or not with As^3+^ exposure.

## Limitations of the study

An important question that remains unanswered in this study is how comprehensive is of the metabolomic discoveries from the cells treated with the environmentally relevant concentration of As^3+^. It has to be recognized that there are some intrinsic limitations in the current technologies in both untargeted and targeted metabolomics 31. First, some dynamic changes of metabolites in response to As^3+^ might be unidentified due to limits of detection sensitivity on some unique chemical structures and physicochemical properties of the metabolites. Second, the obtained data of metabolomics were particularly on a fraction of metabolites that are based on the established databases with annotable metabolites. In addition, although ChIP-seq data indicated possible roles of As^3+^ on several enzymes that are critical for specific metabolic programs, it is well known that the availability of metabolic precursors and the rate of catabolism of substrate are also determinant factors for the abundance of any given metabolites. Furthermore, the present study focused on metabolomics and partial ChIP-seq only. Future studies should integrate these data with RNA-seq and proteomics investigations to provide insights into the detailed mechanistic understanding of As^3+^ carcinogenesis from epigenetics, gene expression to metabolomics. Despite these limitations, our study significantly expands our knowledge of specific and general changes in the cellular metabolomic landscape in response to environmental As^3+^ exposure.

## Materials and Methods

### Cells culture and Western blotting

The human bronchial epithelial cell line BEAS-2B was purchased from the American Tissue Culture Collection (ATCC, Manassas, VA). The cells were cultured in DMEM (Invitrogen, NY) containing 5% FBS (Invitrogen), 1% penicillin/streptomycin, 1% L-glutamine (Sigma-Aldrich, St. Louis, MO) at 37 ºC humidified incubator with 5% CO_2_. The cells were treated with 0.25 μM arsenic chloride (Sigma-Aldrich) consecutively for 1 to 6 months or 6 to 24 weeks. In short-term culture, the cells were treated with 1 μM As^3+^ for 6h. At the end of each time point, the cells were collected for Western blotting using the indicated antibodies purchased from Cell Signaling Technology (Danvers, MA) or Abcam (Cambridge, MA).

### Soft agar colony formation assay

Six-well cell culture plates containing 2 ml of 0.5% low melting agar (Bio-Rad) in DMEM were set at room temperature for 20 min, followed by adding 1 ml of 0.3% low melting agar containing 3 × 10^3^ cells/ml and incubated at room temperature for another 20 min. Then 2 ml regular cell culture medium was added into each well and the plates were incubated in a humidified incubator at 37 ºC for 2 to 3 weeks. Medium was changed twice a week. Cell colonies were photographed by bright field microscopy at the end of incubation. Colony diameter was measured using scale bar during image documentation.

### Chromatin immunoprecipitation-sequencing (ChIP-Seq)

ChIP-Seq was performed as previously described 16, 32. Briefly, ten million control cells or cells treated with 0.25 μM As^3+^ for 24 weeks were fixed and subjected to immunoprecipitation using ChIP-grade antibodies from Active Motif (Carlsbad, CA) against histone H3 lysine 4 trimethylation (H3K4me3), H3K27me3 and H3K9me3. Genome hg19 was used as a reference in the ChIP-Seq data analysis. ChIP-Seq data were visualized by the UCSC genome browser. ChIP-seq for Nrf2 and HIF1α is as previously described 16.

### Untargeted global metabolomics

Control cells and the cells treated with 0.25 μM As^3+^ for 6, 13, 16, 20, and 24 weeks, six replicates in each group, were prepared for metabolomics analysis. The samples were prepared by using the automated MicroLab STAR® system from Hamilton Company. Several recovery standards were added prior to the first step in the extraction process for QC purposes. To remove protein, dissociate small molecules bound to protein or trapped in the precipitated protein matrix, and to recover chemically diverse metabolites, proteins were precipitated with methanol under vigorous shaking for 2 min followed by centrifugation. The resulting extract was divided into five fractions: two for analysis by two separate reverse phase/ultrahigh performance liquid chromatography-tandem mass spectroscopy RP/UPLC-MS/MS methods with positive ion mode electrospray ionization (ESI), one for analysis by RP/UPLC-MS/MS with negative ion mode ESI, one for analysis by HILIC/UPLC-MS/MS with negative ion mode ESI, and one sample was reserved for backup. Samples were placed briefly on a TurboVap® (Zymark) to remove the organic solvent. The sample extracts were stored overnight under nitrogen before preparation for analysis.

### QA/QC

Several types of controls were analyzed in concert with the experimental samples: a pooled matrix sample generated by taking a small volume of each experimental sample served as a technical replicate throughout the data set; extracted water samples served as process blanks; and a cocktail of QC standards that were carefully chosen not to interfere with the measurement of endogenous compounds were spiked into every analyzed sample that allowed better instrument performance monitoring and aided in chromatographic alignment. Instrument variability was determined by calculating the median relative standard deviation (RSD) for the standards that were added to each sample prior to injection into the mass spectrometers. Overall process variability was determined by calculating the median RSD for all endogenous metabolites present in 100% of the pooled matrix samples. All samples were analyzed by UPLC-MS/MS. Quantification, bioinformatics, and statistical analysis of metabolomics was described in the technical note of Metabolon (Morrisville, NC).

### Statistics and overall survival analysis of the cancer patients

Student's t-test was used for some quantitative experimental results and p < 0.05 was considered statistically significant. Multiple different statistical methods, including one/two-way ANOVA, Hotelling's T^2^ test, etc., were applied for the quality control, data curation and normalization, and bioinformatics analysis of ChIP-seq. Overall survival analysis of the cancer patients were estimated using the online datasets from Kaplan-Meier Plotter.
